# Effect of Glycine on BV-2 Microglial Cells Treated with Interferon-γ and Lipopolysaccharide

**DOI:** 10.3390/ijms21030804

**Published:** 2020-01-26

**Authors:** Florentina Egger, Martin Jakab, Julia Fuchs, Karin Oberascher, Gabriele Brachtl, Markus Ritter, Hubert H. Kerschbaum, Martin Gaisberger

**Affiliations:** 1 Institute of Physiology and Pathophysiology, Paracelsus Medical University, 5020 Salzburg, Austria; florentina.egger@pmu.ac.at (F.E.); martin.jakab@pmu.ac.at (M.J.); julia.fuchs@pmu.ac.at (J.F.); markus.ritter@pmu.ac.at (M.R.); 2 Gastein Research Institute, Paracelsus Medical University, 5020 Salzburg, Austria; 3 Ludwig Boltzmann Institute for Arthritis and Rehabilitation, Paracelsus Medical University, 5020 Salzburg, Austria; 4 Department of Cell Biology, University of Salzburg, 5020 Salzburg, Austria; karin.oberascher@sbg.ac.at (K.O.); hubert.kerschbaum@sbg.ac.at (H.H.K.); 5 Institute for Experimental and Clinical Cell Therapy, Core Facility for Flow Cytometry, SCI-TRECS, Paracelsus Medical University, 5020 Salzburg, Austria

**Keywords:** apoptosis, cell volume, cytokines, glycine, inflammation, microglia, neuroprotection

## Abstract

Microglia are first-line defense antigen-presenting phagocytes in the central nervous system. Activated microglial cells release pro-inflammatory cytokines and can trigger an oxidative burst. The amino acid glycine exerts anti-inflammatory, immunomodulatory and cytoprotective effects and influences cell volume regulation. This study aimed to investigate the role of glycine in the modulation of inflammatory processes in mouse BV-2 microglial cells. Inflammatory stress was induced by lipopolysaccharide/interferon-γ (LPS/IFN-γ) treatment for 24 h in the absence or presence of 1 or 5 mM glycine. Cells were analyzed by flow cytometry for cell volume, side scatter, apoptosis/necrosis and expression of activation-specific surface markers. Apoptosis progression was monitored by life cell imaging. Reduced glutathione/oxidized glutathione (GSH/GSSG) ratios and release of the pro-inflammatory cytokines IL-6 and TNF-α were measured using luminescence-based assays and ELISA, respectively. We found that LPS/IFN-γ-induced apoptosis was decreased and the fraction of living cells was increased by glycine. Expression of the surface markers CD11b, CD54 and CD80 was dose-dependently increased, while IL-6 and TNF-α release was not altered compared to LPS/IFN-γ-treated cells. We showed that in BV-2 microglial cells glycine improves viability and counteracts deleterious responses to LPS/IFN-γ, which might be relevant in neurodegenerative processes associated with inflammation, like Alzheimer’s or Parkinson’s disease.

## 1. Introduction

Microglial cells are antigen-presenting phagocytes in the central nervous system (CNS) and build the first line and main form of active immune defense in the CNS against injury and infection. There are two major phenotypes of microglia, the non-activated ramified form (i.e., the “resting state”) and the activated form. Non-activated microglial cells are not resting but rather build a network to stay in contact with other microglia cells, interact with neighboring neurons and continuously scan the environment with their filopodia. If alterations are recognized, they change their morphology into an active, round-shaped form able to migrate, proliferate or phagocytose [1]. Active microglia can differentiate to M1 and M2 phenotypes, where M1 enhance cytotoxic and M2 trigger neuroprotective effects [2]. As a result, activated microglial cells perform two main functions: they support damaged or endangered neurons by supplying neurotrophic and anti-inflammatory factors, and they release pro-inflammatory cytokines that can induce an oxidative burst against pathogens. However, by this inflammatory response healthy host cells can also be damaged and microglia themselves must handle increasing oxidative stress [3]. Furthermore, microglial cells push the immune response by expressing specific surface markers and intracellular enzymes [4] such as complement receptors, pattern recognition receptors (important for the recognition of pathogen molecules), cytokine receptors and receptors for the activation of the adaptive immune system [5]. As a final consequence of inflammation and oxidative stress, cells may undergo apoptosis or necrosis. The elimination of activated microglia by programmed cell death has been postulated to be essential to maintain homeostasis and physiological function in the CNS [6].

The non-essential amino acid glycine is essential for protein synthesis and metabolism and plays a major role in cellular homeostasis and anti-inflammation in many organs and tissues. Dietary glycine e.g., protected rodents from post-operative inflammation after an abdominal surgery of ileus [7] and protected mouse hepatic tissue from damage after lipopolysaccharide (LPS) injection [8]. Compared to its well-known functions as a neurotransmitter, the knowledge on effects of glycine in brain tissue and specifically on microglia is sparse. Importantly, glycine has recently been found to inhibit the pro-inflammatory activity in microglial cells after ischemia-reperfusion injury by promoting M2 polarization through protein kinase B (PKB, Akt)-mediated inhibition of NF-κB p65 and Hif-1α [9]. We have previously shown that in BV-2 microglial cells and primary mouse microglia glycine can act as a chemoattractant to stimulate migration and that glycine-uptake via sodium-dependent neutral amino acid transporters (SNATs) stimulates phagocytosis [10,11].

Based on the evidence that glycine can modulate inflammatory processes, this study was performed to find out if glycine affects LPS/ interferon-γ (IFN-γ)-induced inflammatory cell stress and viability in BV-2 microglial cells.

## 2. Results

### 2.1. Analysis of Apoptosis and Necrosis Using Flow Cytometry

**Figure 1:**
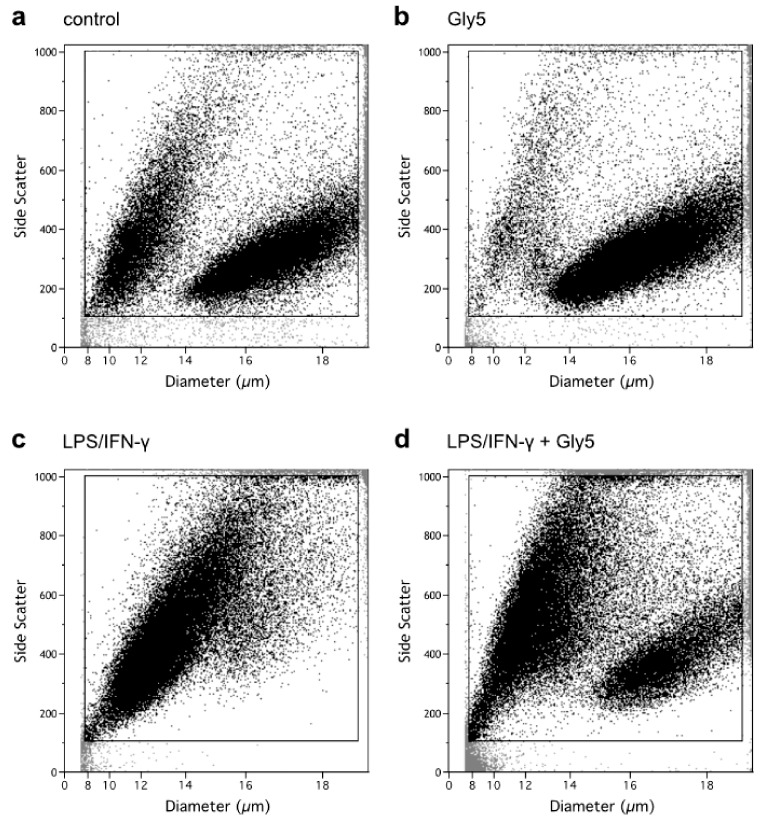
Side scatter vs. cell diameter (µm) plots of (**a**) untreated BV-2 cells, (**b**) cells treated with 5 mM glycine (Gly5), (**c**) cells treated with lipopolysaccharide/interferon-γ (LPS/IFN-γ) alone and (**d**) cells treated with both LPS/IFN-γ and 5 mM glycine (Gly5) after 24 h of incubation. Cell counts were gated to exclude cell debris. One experiment out of six independent experiments is shown.

To investigate the effect of LPS/IFN-γ treatment in the absence and presence of glycine on markers of cell death, flow cytometry was performed to assess cell diameter/volume and cell granularity (side scatter) as well as FITC-annexin-V (ANN-V) and 7-amino-actinomycin D (7-AAD) staining. Two BV-2 cell populations differing in diameter/volume and side scatter can be discriminated (Figure 1): one population of higher mean cell volumes (MCV) of 651 ± 8 fl (right population) and one of smaller MCV of 256 ± 7 fl (left population). Without treatment (control, Figure 1a), a higher percentage of cells (87 ± 4%) was present in the right than in the left area. In the presence of 5 mM glycine, the percentage of cells in the right population increased to 93 ± 1% (Figure 1b). Upon treatment with LPS/IFN-γ alone, the right population nearly became extinct, while the left population showed an accrual to 75 ± 8% and an increased side scatter signal (Figure 1c), indicating cell shrinkage (apoptotic volume decrease, AVD [12]) and increased cell granularity as hallmarks of apoptosis. Co-incubation with LPS/IFN-γ and 5 mM glycine partially prevented this LPS/IFN-γ-triggered shift of cells from the right to the left population with the latter accounting for 54 ± 12% of cell counts (Figure 1d).

**Figure 2:**
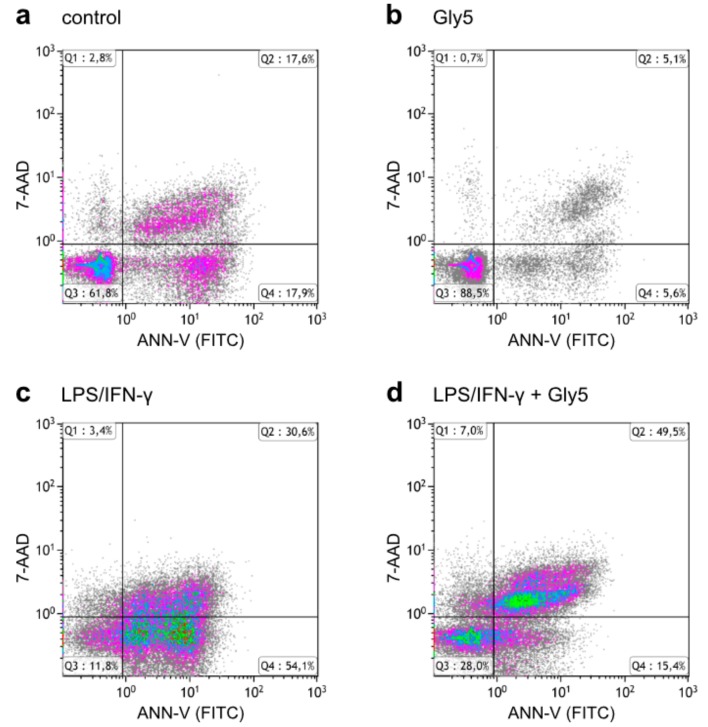
7-AAD vs. FITC-ANN-V density plots of (**a**) untreated BV-2 cells, (**b**) cells treated with 5 mM glycine (Gly5), (**c**) cells treated with LPS/IFN-γ alone and (**d**) cells treated with both LPS/IFN-γ and 5 mM glycine (Gly5) after 24 h of incubation. Cell counts were gated to exclude cell debris. One experiment out of four independent experiments is shown.

To further study the effects of LPS/IFN-γ and glycine on viability and apoptosis, cells were analyzed for FITC-ANN-V and 7-AAD staining and grouped into living cells (ANN-V-/7-AAD-; Quadrant (Q)3), early apoptotic cells (ANN-V+/7-AAD-; Q4), late apoptotic cells (ANN-V+/7-AAD+; Q2) and necrotic cells (ANN-V-/7-AAD+; Q1), as shown in Figure 2.

**Figure 3:**
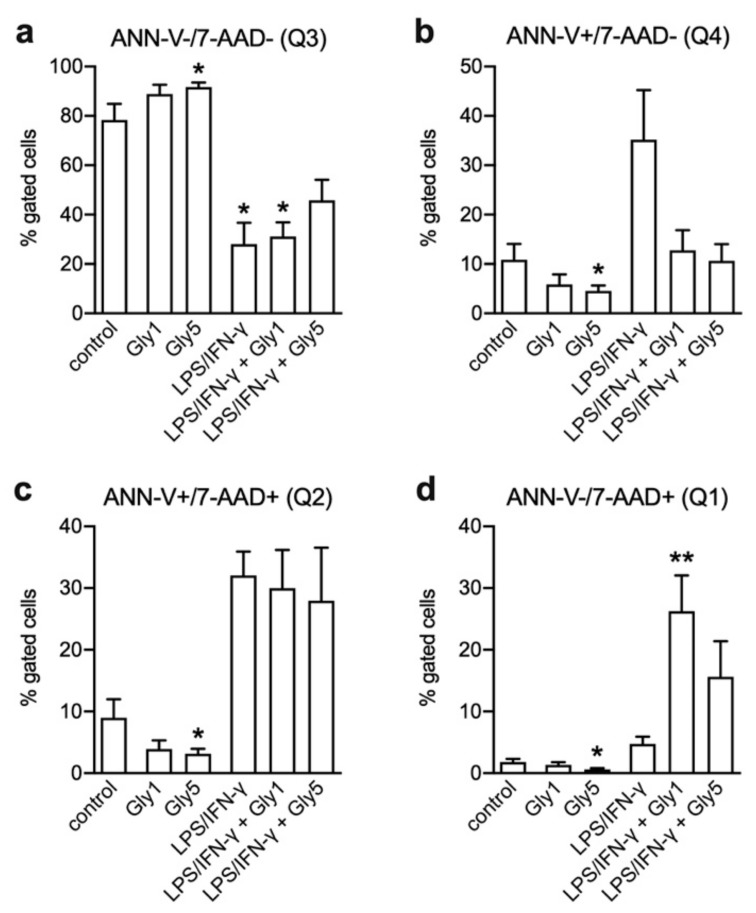
Determination of apoptotic cell fractions in BV-2 cells after 24 h without treatment (control), after incubation with 1 or 5 mM glycine (Gly1, Gly5), with LPS/IFN-γ alone as well as with LPS/IFN-γ and 1 or 5 mM glycine. (**a**) Living cells (ANN-V-/7-AAD-; Q3), (**b**) early apoptotic cells (ANN-V+/7-AAD-; Q4), (**c**) late apoptotic cells (ANN-V+/7-AAD+; Q2) and (**d**) necrotic cells (ANN-V-/7-AAD+; Q1). Asterisks indicate significances compared to control (* *p* < 0.05; ** *p* < 0.01). Four independent experiments were performed per treatment.

Under control conditions, the fraction of living cells in Q3 was 78.3 ± 6.5% and increased in the presence of 1 and 5 mM glycine to 88.9 ± 3.7% and 91.7 ± 1.9%, respectively (Figure 3a). This was accompanied by reduced fractions of cells in Q4 (early apoptotic), Q2 (late apoptotic) and Q1 (necrotic), as apparent also in Figure 2b showing the effect of 5 mM glycine in one of six representative experiment. In controls, the respective fractions comprised 10.9 ± 3.2% (Q4), 9.0 ± 3.0% (Q2) and 1.8 ± 0.5% (Q1). In the presence of 5 mM glycine these values were reduced by ~6.4%, ~5.8% and ~1.2%, respectively (Figure 3b–d).

Treatment for 24 h with LPS/IFN-γ alone diminished the fraction of living cells (Q3) by almost two-thirds to 28.0 ± 8.7% while the percentages of cells in Q4, Q2 and Q1 increased to 35.2 ± 10.0%, 32.1 ± 3.9% and 4.7 ± 1.2%, respectively. Figure 2c shows this shift of the living cell fraction towards early and late apoptosis. Co-incubation with glycine dose-dependently mitigated the pro-apoptotic effect of LPS/IFN-γ. Compared to LPS/IFN-γ treatment alone, the reduction of living cells was ~18% less with 5 mM glycine (to 45.8 ± 8.3%) and both 1 and 5 mM glycine caused a drop in the percentage of early apoptotic cells in Q4 to less than one-third (12.8 ± 4.1% and 10.6 ± 3.4%, respectively) (Figure 3b). For the effect of 5 mM glycine see Figure 2d vs. Figure 2c. While the percentage of late apoptotic cells in Q2 was only slightly reduced by glycine (Figure 3c), the fraction of cells in Q1 was even higher in cells co-treated with 1 and 5 mM glycine (26.2 ± 5.8% and 15.6 ± 5.8%, respectively) than in cells treated with LPS/IFN-γ alone (Figure 3d).

### 2.2. Live-Cell Imaging

**Figure 4:**
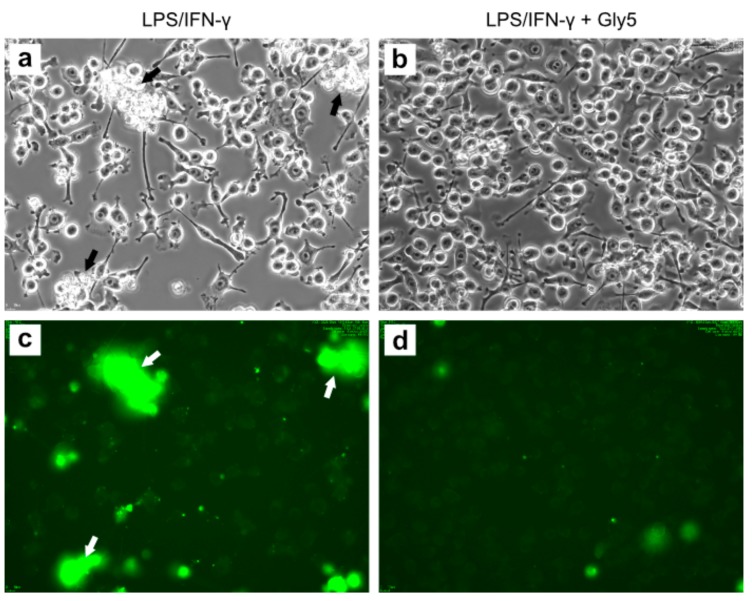
Phase-contrast images (**a**,**b**) and corresponding fluorescence images of FITC-ANN-V stained (**c**,**d**) BV-2 cells treated for 24 h with LPS/INF-γ alone (**a**,**c**) or in the presence of 5 mM glycine (**b**,**d**). Magnification 20×. Arrows indicate aggregates of cells which are already apoptotic, or which are about to collapse.

To further study the effect of glycine on LPS/IFN-γ-induced apoptosis, we analyzed the behavior and morphology of FITC-ANN-V-labeled BV-2 cells in time-lapse experiments over 24 h. Figure 4 shows phase-contrast images (upper panels) and corresponding fluorescence images (lower panels) after 24 h of treatment with LPS/INF-γ in the absence (left panels) and presence of 5 mM glycine (right panels). Cells treated with LPS/IFN-γ alone were generally smaller and possessed short filopodia or even lacked them. Clusters of cells with apoptotic morphology and apoptotic bodies were frequently observed (Figure 4a). Corresponding to cells morphologically identified as being apoptotic or dead, fluorescence imaging (Figure 4c) revealed ANN-V+ cells at these positions (indicated by arrows). In contrast, cells cultured in the presence of both LPS/IFN-γ and 5 mM glycine appeared larger and they extended long filopodia (Figure 4b). The corresponding fluorescence images showed only a small number of ANN-V+ cells with faint staining, indicating that apoptotic events were scarcer (Figure 4d).

### 2.3. Microglia Activation Marker Analysis

**Figure 5:**
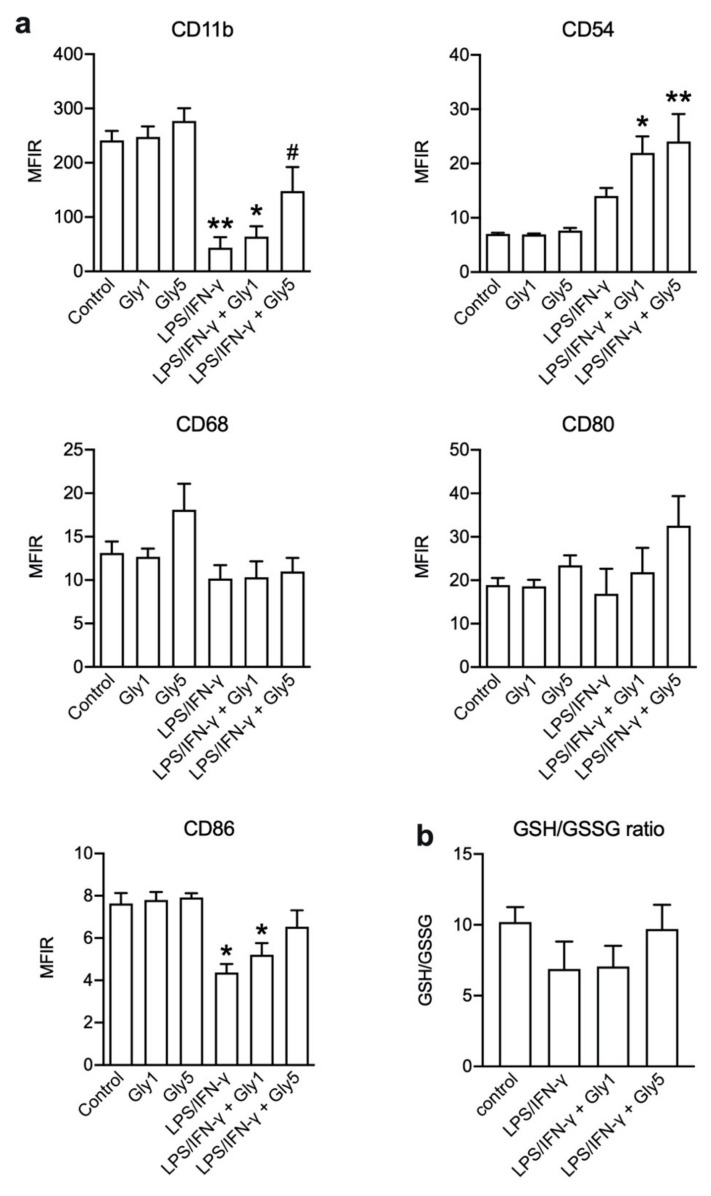
(**a**) Median fluorescence intensity ratios (MFIR) of CD11b, CD54, CD68, CD80 and CD86 staining in BV-2 cells without treatment (control) and treated for 24 h with 1 or 5 mM glycine (Gly1, Gly5) or for 24 h with LPS/IFN-γ in the absence and presence of 1 or 5 mM glycine (Gly1, Gly5), respectively. Asterisks indicate significances compared to control (* *p* < 0.05; ** *p* < 0.01); the hash indicates significance compared to LSP/IFN-γ treatment (# *p* < 0.05). Six independent experiments were performed per treatment. (**b**) Reduced glutathione/oxidized glutathione (GSH/GSSG) ratios measured under the same conditions as in (a). Three independent experiments were performed per treatment.

Cell surface expression of activation markers CD11b, CD53, CD68, CD80 and CD86 [3] was determined in unstimulated cells, cells incubated for 24 h in the presence of 1 or 5 mM glycine, cells stimulated with LPS/IFN-γ alone as well cells co-treated with LPS/IFN-γ and 1 or 5 mM glycine (Figure 5a).

Under 1 or 5 mM glycine alone, no changes in CD11b, CD54, CD80 and CD86 expression were evident compared to controls, while for CD68 the median fluorescence intensity ratio (MFIR) was increased from 13.1 ± 1.3 in untreated cells to 18.1 ± 3.0 under 5 mM glycine. CD11b showed high expression in untreated cells (MFIR = 241.0 ± 17.4), which decreased under stimulation by LPS/IFN-γ (MIFR = 43.4 ± 19.4). In the presence of 5 mM glycine, this effect was diminished (MIFR = 147.8 ± 44.4). Compared to untreated controls (MIFR = 7.0 ± 0.2), CD54 expression was higher in LPS/IFN-γ-treated cells (MIFR = 14.0 ± 1.5). In the combined presence of LPS/IFN-γ and 1 or 5 mM glycine, CD54 expression was further increased (MIFR = 21.9 ± 3.0 and 24.1 ± 5.1, respectively) compared to control. A dose-dependent effect was observed for CD80 expression with an increase under stimulation with LPS/IFN-γ in combination with 1 or 5 mM glycine (MFIR = 21.9 ± 5.6 and 32.6 ± 6.8, respectively) compared to controls (MIFR = 18.9 ± 1.6). CD86 expression was highest under control conditions (MIFR = 7.6 ± 0.5) and lowest under LPS/IFN-γ treatment (MIFR = 4.4 ± 0.4). In combination with 1 and 5 mM glycine under LPS/IFN-γ treatment, the expression increased again (MFIR = 5.2 ± 0.6 and 6.5 ± 0.8, respectively). No obvious changes were detected for CD68 expression.

### 2.4. Quantification of GSH/GSSG Ratios

The ratio of reduced (GSH) and oxidized glutathione (GSSG) (Figure 5b) was highest under control conditions (GSH/GSSG = 10.2 ± 1.1) and lowest under LPS/IFN-γ treatment (GSH/GSSG = 6.9 ± 1.5). In combination with 1 mM glycine the ratio was in the range of LPS/IFN-γ treatment alone (GSH/GSSG = 7.1 ± 1.5) and increased to control levels under 5 mM glycine (GSH/GSSG = 9.7 ± 1.7).

### 2.5. Release of IL-6 and TNF-α

**Figure 6:**
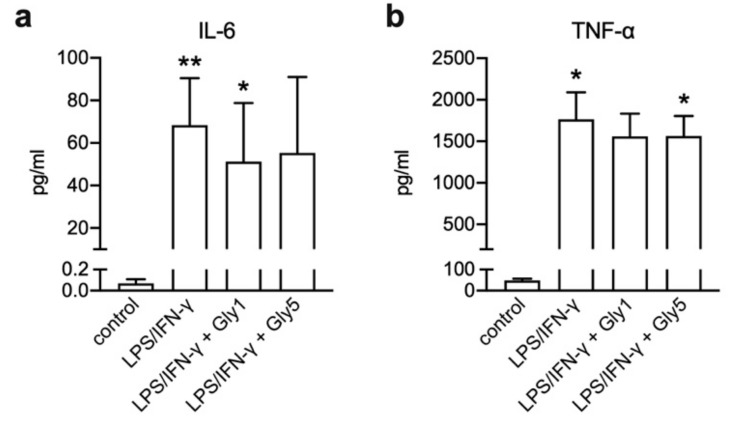
Release of (**a**) IL-6 and (**b**) TNF-α upon LPS/IFN-γ treatment for 24 h in the absence and presence of 1 or 5 mM glycine. Asterisks indicate significances compared to control (* *p* < 0.05; ** *p* < 0.01). Seven independent experiments were performed per treatment.

Pro-inflammatory cytokines IL-6 and TNF-α were measured in BV-2 cell culture supernatants of untreated cells and cells stimulated with LPS/IFN-γ in the absence and presence of 1 and 5 mM glycine after 24 h of incubation (Figure 6).

LPS/IFN-γ stimulation caused an increase of IL-6 and TNF-α release (68.3 ± 22.2 pg/mL and 1766 ± 325 pg/mL, respectively) compared to controls (0.1 ± 0.0 pg/mL and 47.8 ± 9.0 pg/mL, respectively). IL-6 secretion in LPS/IFN-γ-stimulated cells in combination with 1 and 5 mM glycine was increased compared to controls (51.2 ± 27.6 pg/mL and 55.29 ± 35.7 pg/mL, respectively) (Figure 6a) and also the concentration of TNF-α in supernatants was increased after LPS/IFN-γ treatment in the presence of 1 or 5 mM glycine (1559 ± 325 pg/mL and 1565 ± 239 pg/mL, respectively) compared to controls (Figure 6b).

## 3. Discussion

Microglial cells constitute the active immune defense in the CNS and are involved in inflammatory and neurodegenerative processes. Their functions are fine-tuned by auto- and paracrine signals [3]. In previous studies, we could show that the immunomodulatory, anti-inflammatory amino acid glycine stimulates phagocytosis in microglial cells [10], and acts as a chemoattractant to stimulate migration [11]. The effects of glycine were inhibited by MeAIB, a competitive inhibitor of Na^+^-coupled neutral amino acid transporters (SNATs), but not by the GlyR blocker strychnine, indicating that glycine uptake rather than its action as a neuro/gliotransmitter underlies these effects [10,11]. Na^+^-coupled uptake of glycine was accompanied by a depolarization of the cell membrane potential and cell swelling, which is followed by activation of volume-regulated anion channels (VRACs) [11] to drive regulatory volume decrease (RVD) [13]. We could also show that inhibition of these channels suppresses the formation of engulfment pseudopodia and phagocytosis in primary mouse microglia and BV-2 cells [14]. VRACs may also contribute to ROS-mediated glutamate release by microglial cells [15], implying that microglial function and brain function in general [16] is tightly linked to cell volume regulatory mechanisms.

The present study was performed to reveal to what extent glycine affected the viability, activation state and secretory activity of microglial cells during inflammation. BV-2 cells were stressed with a combination of LPS and IFN-γ over 24 h. LPS is the most common pro-inflammatory stimulus and causes inflammatory reactions in primary microglia and BV-2 cells [17,18], and IFN-γ is released during CNS injury [18,19,20]. Approximately 90% of the genes induced in BV-2 cells after LPS exposure were also found in primary microglia and showed similar reaction patterns [21], which warrants this cell line as a suitable experimental model. In our experiments, BV-2 cells responded to LPS/IFN-γ stimulation by releasing the pro-inflammatory cytokines IL-6 and TNF-α. This release was only slightly reduced under co-treatment with glycine. However, as we measured the cumulative release over 24 h, differences at earlier or later time points might have been masked.

Although BV-2 cells are notably resistant to the influence of oxidants and inflammatory mediators, LPS-derived protein oxidation induces protein degradation and structural changes in microglia and finally leads to apoptosis or necrosis [22]. Flow cytometric analysis of side scatter vs. electronic volume revealed two distinct populations of BV-2 cells. In LPS/IFN-γ-treated samples the right population (larger cell volume) nearly disappeared or shifted over to the left population, which indicates that cells became smaller and possibly started getting apoptotic. When LPS/IFN-γ treatment was combined with 5 mM glycine, the distribution of cells was similar to the untreated control sample. Of note, treatment with glycine alone showed fewer cells in the left population and a higher percentage of larger cells in the right population compared to the untreated control, indicating a glycine-only effect on cell volume and size. This effect was also visible in LPS/IFN-γ- plus glycine-treated cell samples and might be due to sodium-coupled glycine uptake via SNAT which causes cell swelling [10,23] and counteracts AVD.

ANN-V and 7-AAD staining revealed that the factors cell diameter or volume did not strictly indicate whether BV-2 cells were viable or dead. Even though in the LPS/IFN-γ-treated samples the majority of cells were allocated to the left population with smaller diameter, it also contained living cells and not only apoptotic or necrotic cells and cell debris, as might be assumed. This indicates that both populations most likely contained different phenotypes of BV-2 cells and that the observed volume decrease does not mean in general that these cells are apoptotic. The percentage of living cells was reduced under LPS/IFN-γ treatment compared to the untreated controls from ~80% to ~30%. It has been published that the combination of LPS and IFN-γ treatment led to massive cell death in primary microglial cells. This effect was based on the activation of caspase-2, caspase-3 and caspase-7, and other pro-apoptotic pathways [24]. With respect to our study, this relates to the fractions of early and late apoptotic cells, which increased after LPS/IFN-γ treatment compared to untreated cells. Glycine co-treatment counteracted the LPS/IFN-γ effect and decreased the percentage of early apoptotic cells, while the fraction of late apoptotic cells was only slightly reduced, and the percentage of necrotic cells was even increased. This apparent increase in necrotic cells might have been due to a delay in the progression of LPS/IFN-γ-induced cell death by glycine, so that after 24 h a higher percentage of cells was found in Q1 compared to LPS/IFN-γ treatment alone, where cells might have already disintegrated and shifted to the debris and therefore were excluded from analysis. This result might also be partly attributed to the settings chosen for analysis; since cell populations could hardly be discriminated in LPS/IFN-γ-treated samples, quadrant region settings were based on untreated control samples. In samples co-treated with LPS/IFN-γ and glycine, this could have led to an overestimation of necrotic cells showing late apoptotic cells in Q1.

The inhibitory or delaying effect of glycine on LPS/IFN-γ-mediated apoptosis is also supported by live-cell imaging showing higher fluorescence signals in LPS/IFN-γ-treated cells than in cells co-treated with 5 mM glycine. Moreover, phase-contrast images showed more ramified and larger cells and less apoptotic cells after glycine co-treatment compared to LPS/IFN-γ-only treated cells.

We found that LPS/IFN-γ-induced cell death in BV-2 cells was mitigated at glycine concentrations of 1 and 5 mM. The normal plasma concentration of glycine is 0.25–0.5 mM [25], so the effective concentrations in our cell system seem high. However, during neuronal activity peak glycine concentrations of 2.2–3.5 mM have been determined in glycinergic synapses [26], showing that in the nervous system glycine reaches concentrations above plasma levels under physiological conditions. Astrocytes play a major role in glycine uptake from the synaptic cleft, and store glycine at concentrations up to 3–6 mM [27]. In case of traumata, stroke, ischemia/reperfusion injury or neurodegeneration, when dying neurons and glial cells release stored glycine and other neutral amino acids that are transported via SNATs (e.g., glutamine or proline) into the restricted interstitial space, these compounds might accumulate to reach concentrations in the millimolar range. Moreover, elevated glycine levels both in blood and cerebrospinal have been reported from metabolic disorders like non-ketotic hyperglycinemia [28,29] and inflammatory diseases such as meningitis [30] and multiple sclerosis [31].

Cluster of differentiation (CD) antigens play an important role in normal cellular function of macrophages and show different expression patterns during inflammatory responses and cell stress. Macrophage types like M1 and M2 and their subclasses show unique surface marker expression profiles. In the present study, CD11b, CD54, CD80 and CD86 were detected by flow cytometry as indicators of cell activation status, antigen presentation and homing to sites of lesion or damage. In contrast, the exact function of CD68 in macrophages is poorly investigated. There is a fast turnover between highly expressed intracellular CD68 on late endosomes and the plasma membrane. CD68 is known to bind to oxidized LDL, phosphatidylserine and apoptotic cells. It seems to be involved in cell adhesion and antigen presentation, and acts as a scavenger receptor [32,33]. The analyzed surface marker pattern indicates that under 5 mM glycine, in combination with LPS/IFN-γ, cells are in an activated, immunological regular status and less prone to programmed cell death. CD68 expression was not altered, except from an upregulation under 5 mM glycine alone compared to untreated controls. Expression of all other measured surface markers was unaffected by 1 or 5 mM glycine alone.

For the accumulation at lesion sites, microglial cells need to express surface antigens like CD11b (integrin alpha M) and CD54. CD11b is part of the heterodimeric integrin alpha-M beta-2 molecule and is important for intracellular cell adhesion and migration as a binding protein for CD54 and other proteins in microglia. It is one of the potential markers of microglial activation and is expressed in LPS-, IFN-γ- and ROS-stimulated cells in response to nitric oxide (NO) [34]. CD11b MFIR was drastically lowered under LPS/IFN-γ stimulation and a dose-dependent increase in combination of inflammatory treatment and 1 and 5 mM glycine was observed. Cells under LPS/IFN-γ treatment seem to lower the expression of special surface markers like CD11b, perhaps due to the onset of programmed cell death resulting in a loss of cell function upon such strong inflammatory stimulation. Glycine seems to prolong a “normal” CD11b cell surface marker status and thereby enhance microglial immune functions such as migration and phagocytosis.

The cell adhesion glycoprotein CD54, also known as intercellular adhesion molecule 1 (ICAM-1), is expressed on activated macrophages. It gets upregulated by inflammatory stimuli and pro-inflammatory cytokines, hormones, pathogens or oxidative stress and interacts with CD11a and CD11b. The latter are important for endothelial cell binding and migration, induction of an inflammatory response and stimulation of T-cell activation [35]. Downregulation of CD54 is associated with endotoxin tolerance and a protective M2 microglia phenotype [36]. CD54 expression was higher under LPS/IFN-γ treatment in combination with 1 or 5 mM glycine. This indicates that under inflammatory stimulation, glycine might promote a pro-inflammatory status of BV-2 cells.

The costimulatory factors CD80 (B7.1) and CD86 (B7.2) play a crucial role in infiltrated T-cell activation, antigen presentation and neuroinflammation [37,38,39]. Blocking of the costimulatory signal can suppress autoimmunity and inflammation, even causing an anti-inflammatory profile. Its expression was also shown without T-cell infiltration, which indicates a broader role in inflammation in the CNS [40]. In our experiment, CD80 surface expression was highest under inflammatory stimulation in combination with 5 mM glycine and analysis of CD86 expression showed that its downregulation under LPS/IFN-γ treatment was diminished by 5 mM glycine.

Summing up, our data shows that glycine given together with LPS/IFN-γ stimulation alters the surface marker expression of microglia cells, compared to LPS/IFN-γ stimulation alone. Microglia phenotypes, like classically activated pro-inflammatory M1 cells or alternatively activated growth-promoting M2 microglia, show a different surface marker pattern [41,42]. The higher CD80 and CD86 expression in combination with the reduced CD11b expression suggests that in our setup glycine keeps microglial cells in a classically activated pro-inflammatory state.

The ratio of reduced and oxidized glutathione (GSH/GSSG) is a measure of oxidative cell stress. Under normal conditions, 90% of glutathione is available as reduced GSH [43]. Our results show that 5 mM glycine can increase the ratio from ~70% under LPS/IFN-γ back to over 90%, indicating reduced oxidative stress in BV-2 under glycine treatment.

## 4. Materials and Methods

### 4.1. Cell Culture and Substances

The immortal cell line BV-2 (Interlab Cell Line Collection Genova, Genoa, Italy) was grown in RPMI 1640 Medium (Biochrom by Merck, Darmstadt, Germany) containing 10% fetal bovine serum (FBS; Biochrom) and 1% antibiotic-antimycotic solution (ABAM; Sigma Aldrich, Darmstadt, Germany) at 37 °C, 5% CO_2_ and 95% air (standard culture conditions). Subcultures were established once a week by trypsin/EDTA treatment (0.25%; Sigma Aldrich).

For the experiments, cells were seeded in 3.5 cm^2^ culture dishes at 0.6 × 10^6^ cells per dish or in 96-well cell culture plates at 0.11 × 10^5^ cells per well and cultured overnight. Cells were then incubated for 24 h in Minimum Essential Medium (MEM) without FBS (untreated controls) or serum-free MEM containing LPS (1 µg/mL, Sigma Aldrich) and IFN-γ (100 units/mL, Thermo Fischer Scientific, Vienna, Austria) with or without 1 or 5 mM glycine (Sigma Aldrich) (standard treatment).

### 4.2. Flow Cytometry

BV-2 cells were grown in 3.5 cm^2^ culture dishes under standard culture conditions. After standard treatment, supernatants were collected and cells were harvested using trypsin/EDTA. The supernatants and the detached cells were centrifuged together for 5 min (230× *g*). Cell pellets were resuspended in flow cytometry buffers for further analysis (Biozym, Hessisch Oldendorf, Germany and eBioscience by Thermo Fisher Scientific).

For apoptosis and necrosis characterization, cells were stained with FITC-Annexin-V (ANN-V) and 7-amino-actinomycin D (7-AAD) (Biolegend, San Diego, CA, USA) according to the manufacturer’s protocol. Measurements were performed on a Cell Lab Quanta^TM^ SC flow cytometer (Beckman Coulter, Krefeld, Germany). Debris (particles with a diameter < 7 μm) and cell aggregates (diameter > 20 μm) were excluded from analysis and 20,000–50,000 single cells were analyzed per sample. Dyes were excited with a 488-nm argon laser and fluorescence emissions were measured at 525 nm and 670 nm. Unstained and single-stained samples were used for photo multiplyer tube (PMT) voltage setting and the compensation of FITC overlap in the 7-AAD channel. Cell diameters (in µm) and mean cell volume (MCV in fl) were directly measured using the Coulter principle. The electronic volume (EV) channel was calibrated using 10-μm Flow-Check fluorospheres (Beckman Coulter).

For surface marker detection, cell pellets were incubated with a fluorescent multistain containing antibodies against CD54-FITC, CD11b-APC-eFluor^®^780, CD68-PE-Cy7, CD80-PE, CD86-eFluor^®^450 or the matching isotype controls (Thermo Fisher Scientific). The isotype controls are non-specific antibodies of the same hosts and antibody subtypes with the same fluorochromes. The fluorescence signal of the primary antibodies and the related isotype control was used to calculate the median fluorescence intensity ratio (MFIR) of the fluorescence values for each sample. The MFIR was calculated by dividing the median fluorescence values of the antibodies by the median fluorescence values of the isotype control. Samples were measured on a BD LSRFortessa™ flow cytometer (BD, Heidelberg, Germany) and analyzed with Kaluza Analysis 1.3 Software (Beckman-Coulter).

### 4.3. Live-Cell Imaging

BV-2 cells were grown under standard culture conditions. During treatment with LPS/IFN-γ or LPS/IFN-γ + 5 mM glycine in the presence of FITC-ANN-V, cells were kept the in a Nikon BioStation IM at 37 °C, 5% CO_2_ and 95% air and corresponding phase-contrast and fluorescence (λ_ex_ 488 nm, λ_em_ 525 nm) images were captured every 15 min over 24 h.

### 4.4. Quantification of Total Glutathione Ratios

BV-2 cells were grown in 96-well cell culture plates under standard culture conditions. After standard treatment, the luminescence-based non-lytic GSH/GSSG-Glo^TM^ assay (Promega, Mannheim, Germany) was performed and GSH and GSSG ratios (GSH/GSSG) were calculated according to the manufacturer’s protocol.

### 4.5. IL-6 and TNF-α ELISA

BV-2 cells were seeded in 3.5 cm^2^ culture dishes and grown under standard culture conditions. After standard treatment, supernatants (2 × 500 μL per sample) were collected in 1.5 mL Eppendorf tubes and stored frozen at −20 °C until usage. TNF-α and IL-6 protein in the supernatants were measured with an ELISA Ready-SET-Go^®^ Kit (Invitrogen by Thermo Fisher Scientific) according to the manufacturer’s instructions. Samples were measured at 450 nm with a multimode reader (Tecan Spark^®^, Grödig, Austria).

### 4.6. Statistics

Data are expressed as mean ± standard error of mean (sem) and all calculations were performed using GraphPad Prism 8 software (GraphPad Software, La Jolla, CA, US). The non-parametric Friedman test combined with Dunn’s test for multiple comparisons was used. Means were considered to be significantly different at *p*-values < 0.05. Significances compared to the untreated control group and LPS/IFN-γ treatment are indicated as asterisks (*) and hashes (#), respectively. At least three independent experiments were performed per treatment.

## 5. Conclusions

Summarizing our findings, this study shows that glycine improves the viability of BV-2 microglial cells, mitigates the pro-apoptotic effect of LPS/IFN-γ treatment and reduces oxidative stress. While microglial cells play an active role in inflammation by releasing pro-inflammatory cytokines, the protective effects of glycine along with its stimulatory effects on migration and phagocytosis, as previously shown, might add to ensuring microglial function in the defense against brain injury during inflammation.

## Abbreviations

7-AAD	7-amino-actinomycin D
ANN-V	Annexin-V
AVD	Apoptotic volume decrease
CNS	Central nervous system
ELISA	ELISA, enzyme linked immunosorbent assay
GlyR	Glycine receptor
GSH/GSSG	Reduced/oxidized glutathione
IL-6	Interleukin-6
IFN-γ	Interferon-γ
LPS	Lipopolysaccharide
MCV	Mean cell volume
MeAIB	α-(Methylamino)isobutyric acid
MFIR	Median fluorescence intensity ratio
NO	Nitric oxide
ROS	Reactive oxygen species
RVD	Regulatory volume decrease
SNAT	Sodium-dependent neutral amino acid transporter
TNF-α	Tumor necrosis factor-α
ROS	Reactive oxygen species
VRAC	Volume-regulated anion channel/current
